# Reported methodological quality of medical systematic reviews: Development of an assessment tool (ReMarQ) and meta-research study

**DOI:** 10.1017/rsm.2024.14

**Published:** 2025-03-07

**Authors:** Manuel Marques-Cruz, Rafael José Vieira, Daniel Martinho-Dias, José Pedro Barbosa, António Cardoso-Fernandes, Francisco Franco-Pêgo, Paula Perestrelo, Sara Gil-Mata, Tiago Taveira-Gomes, José Miguel Pêgo, João A. Fonseca, Luís Filipe Azevedo, Bernardo Sousa-Pinto

**Affiliations:** 1 MEDCIDS – Department of Community Medicine, Information and Health Decision Sciences, Faculty of Medicine, University of Porto, Porto, Portugal; 2 CINTESIS@RISE – Health Research Network, University of Porto, Porto, Portugal; 3 Public Health Unit Douro I, ACES Douro I – Marão e Douro Norte, Northern Region Health Administration, Vila Real, Portugal; 4 Family Health Unit Ao Encontro da Saúde, ACES Santo Tirso-Trofa, Trofa, Portugal; 5 Stomatology Department, Centro Hospitalar Universitário de São João, Porto, Portugal; 6 Internal Medicine Department, Hospital of Santa Luzia, Local Health Unit of Alto Minho, Viana do Castelo, Portugal; 7 Central Lisbon University Hospital Centre, Lisboa, Portugal; 8 MTG Research and Development Lab, Porto, Portugal; 9 Faculty of Health Sciences, University Fernando Pessoa (FCS-UFP), Porto, Portugal.; 10 Life and Health Sciences Research Institute (ICVS), School of Medicine, University of Minho, Braga, Portugal.; 11 ICVS/3B’s, PT Government Associate Laboratory, Braga, Portugal

**Keywords:** meta-analysis, methodological quality, reporting quality, systematic reviews

## Abstract

The number of published systematic reviews has increased over the last years, with a non-negligible proportion displaying methodological concerns. We aimed to develop and evaluate a tool to assess the reported methodological quality of medical systematic reviews. The developed tool (ReMarQ) consists of 26 dichotomous items. We applied an item response theory model to assess the difficulty and discrimination of the items and decision tree models to identify those items more capable of identifying systematic reviews with higher reported methodological quality. ReMarQ was applied to a representative sample of medical systematic reviews (excluding those published in the *Cochrane Database of Systematic Reviews*) to describe their methodological quality and identify associated factors. We assessed 400 systematic reviews published between 2010 and 2020, of which 196 (49.0%) included meta-analysis. The most discriminative items were (i) conducting a risk of bias assessment, (ii) having a published protocol and (iii) reporting methods for solving disagreements. More recent systematic reviews (adjusted yearly RR=1.03; 95%CI=1.02 −1.04, *p*<0.001) and those with meta-analysis (adjusted RR=1.34; 95%CI=1.25 −1.43, *p*<0.001) were associated with higher reported methodological quality. Such an association was not observed with the journal impact factor. The items most frequently fulfilled were (i) reporting search dates, (ii) reporting bibliographic sources and (iii) searching multiple electronic bibliographic databases. ReMarQ, consisting of dichotomous items and whose application does not require subject content expertise, may be important (i) in supporting an efficient quality assessment of systematic reviews and (ii) as the basis of automated processes to support that assessment.

## Highlights

### What is already known?

- The increase of published systematic reviews in recent years raises concerns over potential compromises in methodological quality.
- Tools for assessing the reported methodological quality of systematic reviews are currently lacking.

### What is new?

- Our reported methodological quality tool (ReMarQ) measures an important construct, which was incompletely covered by pre-existing frameworks.
- The presence of a risk of bias assessment for individual studies, the existence of a review protocol, and reporting of methods for solving disagreements were identified as key factors to discriminate the overall quality of systematic reviews.
- More recent systematic reviews and those with meta-analysis appear to be associated with higher reported methodological quality as measured by ReMarQ.
- The journal impact factor (and the corresponding percentile) of the journals in which systematic reviews are published do not seem to be associated with their reported methodological quality.

## 1 Introduction

Systematic reviews play a decisive role in the practice of evidence-based medicine.^1^ ^,^ ^2^ Over the last years, the number of published systematic reviews and meta-analyses has increased massively.^3^ This increase may have resulted from several factors: (i) a growth in the number of published primary studies, (ii) an easier access to scientific evidence, and (iii) an increasing pressure to publish.^4^ ^–^ ^6^ However, it is likely that a somewhat large majority of produced systematic reviews and meta-analyses may be misleading and/or contain relevant methodological concerns.^3^ While the full extent of methodological flaws in systematic reviews remains uncertain, it is important to acknowledge that these flaws could potentially have a relevant impact on the findings and conclusions of such reviews.^7^ As an example, previous studies have found that the exclusion of trials published in languages other than English^8^ or the failure to search clinical trial registry databases^9^ may result in the exclusion of eligible primary studies.

The increase in published systematic reviews has been accompanied by the development of tools aiming at improving their quality. Such tools include the preferred reporting items for systematic reviews and meta-analyses (PRISMA) statement,^10^ the risk of bias assessment tool for systematic reviews (ROBIS) ,^11^ and a measurement tool to assess systematic reviews (AMSTAR).^12^ These tools are concerned either with the reporting transparency and completeness of systematic reviews (e.g., PRISMA) or with their risk of bias (e.g., ROBIS). However, there are currently no tools aiming to assess the methodological quality of systematic reviews as reported by their authors (irrespective of whether these result or not in an increased risk of bias). Indeed, this construct (henceforth referred to as ‘reported methodological quality’) is different from both ‘reporting transparency and completeness’ and ‘risk of bias’. The ‘reporting transparency and completeness’ construct—covered by the PRISMA statement—is mostly concerned with whether systematic reviews were reported in an adequate manner and with sufficient detail to allow users to assess the trustworthiness and applicability of the review findings.^13^ As an example, one item of the PRISMA checklist requests that authors present the full search strategies including any filters and limits used.^10^ While this item implies ‘describing any limits applied to the search strategy (such as date or language) and justifying these by linking back to the review’s eligibility criteria’,^13^ it does not distinguish between systematic reviews which report having applied exclusion criteria based on the publication language (less adequate methodological option) vis-à-vis those which report not having applied such criteria (more adequate methodological option).

Similarly, the risk of bias construct, which can be assessed with ROBIS, is more concerned with aspects which may result in an increased probability of biased results. Consequently, (i) important aspects of reported methodological quality of systematic reviews are not present or explicitly stated in the signalling questions of ROBIS (e.g., efforts to avoid double counting of participants) and (ii) subject content and methodologic expertise to complete an assessment are needed.^11^ In contrast, reported methodological quality should cover these important aspects and should not require a specialised background on the question being addressed by the systematic review.

Hence, the main aim of this study was to develop and evaluate the properties of a tool capable of assessing the reported methodological quality of systematic reviews, a construct for which there are no available tools. We also aimed, based on this tool, to assess a representative sample of medical systematic reviews, describing their reported methodological quality and identifying factors potentially associated with such quality.

## 2 Methods

### 2.1 Study design

In this meta-research cross-sectional study, we developed a tool (ReMarQ) to assess the reported methodological quality of systematic reviews and applied it to the Methods section of a random sample of systematic reviews (stratified by the journal citation reports [JCR] category). We used the strengthening of the reporting of observational studies in epidemiology (STROBE) Statement^14^ to guide the reporting of our study. To assess the psychometric properties of ReMarQ, we (i) described the frequency of each item fulfilled, (ii) applied two-parameter logistic item response theory (IRT) models to assess the items’ difficulty and discrimination parameters and (iii) applied classification tree models to identify those items which would more accurately predict the probability that a systematic review would have a higher quality. Additionally, we built regression models to identify variables potentially associated with the reported methodological quality of systematic reviews. The tested variables included the publication year, number of authors, number of references cited, country of the corresponding author’s address, scientific categories, journal impact factor (JIF), and JIF percentile on the year of publication.

### 2.2 Development of the tool

We developed a tool to assess the reported methodological quality of systematic reviews. This tool was conceived as a set of statements (henceforth referred to as ‘items’). In order to define the tool, we started by consulting tools and guidance documents on the reporting completeness (PRISMA 2009 and 2020 Checklists^10^ ^,^ ^15^), methodology (Cochrane Handbook for Systematic Reviews of Interventions^16^), and risk of bias (ROBIS tool^11^) of systematic reviews. We identified a set of statements and practices pertaining reported methodological quality and which were the basis of the items of our tool. These items were developed by consensus, having been written to correspond to a set of dichotomous statements (‘yes/no’ statements, for which ‘yes’ indicated that an item was fulfilled). A pilot version of the tool was first applied to 20 systematic reviews (such an analysis was performed by DMD and BSP independently), being subsequently modified into a final version.

The final version of ReMarQ included 26 items of which 20 were applicable to all systematic reviews and six to only systematic reviews with meta-analysis (Supplementary Tables 2 and 3 display these items mapped to the items from PRISMA and ROBIS tools). We applied ReMarQ to a sample of 400 systematic reviews. For each systematic review, the Methods section was read and the reported methodological quality items were assessed by one examiner (either DMD, JPB, ACF, or FFP) with previous formal training on evidence synthesis (both as part of their undergraduate/medical school syllabus and as part of their postgraduate training [PhD Programme syllabus or formal continuous education courses]) and experience in participating in systematic reviews. In half of the systematic reviews (*n* = 200), a second reviewer (either MMC or RJV) evaluated the provided answers to identify potential misclassifications. Any disagreements were solved by a senior reviewer (BSP). We registered the proportion of systematic reviews that fulfilled each item (we considered an item to be fulfilled whenever it had been classified with an ‘yes’ answer).

For the 20 items assessing the methodology of all of the systematic reviews (i.e., those quality items which did not assess meta-analysis directly), we applied a two-parameter logistic item response theory (IRT) model.^17^ ^–^ ^19^ We replicated this procedure for systematic reviews with meta-analysis with all of the 26 applicable items. The IRT model was applied to study the difficulty and discrimination of each individual item. IRT refers to a set of mathematical models which aim to explain the relationship between a latent variable (in this case, reported methodological quality of systematic reviews) and its observable manifestations (a set of dichotomous items that are manifestations of this latent variable and, consequently, used to evaluate the reported methodological quality of systematic reviews).^17^ In particular, the two-parameter logistic IRT model is a foundational tool in psychometrics and measurement scales in general.^17^ The parameters of the model are: difficulty (*b*), discrimination (*a*) and the latent variable (*θ*):

- Difficulty represents the likelihood of an item being fulfilled, demonstrating that there are items that are more difficult (higher *b*) and more easy (lower *b*) to comply with. In general, this is expressed as the level at which 50% of the units (systematic reviews) sampled is estimated to fulfil a reported methodological quality item.
- Discrimination refers to the ability of an item to differentiate between systematic reviews with different levels of reported methodological quality (higher values of *a* indicate items with greater discrimination power).
- The latent variable represents the underlying ‘reported methodological quality’ of each systematic review, as measured by each item. The two-parameter logistic IRT model assumes unidimensionality and local independence, meaning that it measures only one latent trait and that item classifications are conditionally independent, given the latent variable.^17^

A classification tree model for the 20 ReMarQ items assessing the methodology of all of the systematic reviews was developed with Gini impurity splitting and in order to identify up to seven items which would more accurately predict the probability that a systematic review had at least half of these items fulfilled. As such, we defined a maximum depth for the tree of three nodes and a prior distribution according to the percentage of systematic reviews with and without at least half of its quality items fulfilled to address possible imbalances. Model quality was assessed by computing its kappa coefficient (indicating the agreement between the model-predicted classification and whether the systematic review had at least half of its quality items fulfilled) and its accuracy (indicating the proportion of correct model-predicted classifications). We replicated the same procedure for identifying those quality items predicting that more than two thirds of ReMarQ items would be fulfilled.

Finally, in order to hint at the discriminant validity of our tool, we (MMC, RJV, PP, SGM, and BSP) compared results from ReMarQ against the PRISMA 2020 checklist (as this tool assesses a different construct, namely reporting transparency and completeness) in a random subset of 100 of our included systematic reviews. We did not perform the same analysis against the ROBIS tool, as its ‘users are likely to need both subject content and methodologic expertise to complete an assessment’.^11^

### 2.3 Assessment of the systematic reviews

#### 2.3.1 Eligibility criteria

We included systematic reviews published between 2010 (1 year after the publication of the first PRISMA statement) and 2020 in medical journals indexed in the JCR/Science Edition and with an impact factor. ‘Medical journals’ were defined as those listed in at least one of the 38 categories displayed in Supplementary Table 1 (data collection had begun before Category Groups were available in the JCR). We considered all studies claiming to be ‘systematic reviews’ (henceforth referred to as ‘systematic reviews’) irrespective of the robustness of their methodology. The rationale for this decision was to gather a representative sample of what end users face when coming across an article self-described as a systematic review. Studies claiming to be scoping reviews, rapid evidence reviews, or other types of non-systematic reviews were not included. We opted to exclude articles published in the *Cochrane Database of Systematic Reviews Journal* as its systematic reviews typically display a higher methodological reporting detail, given the more rigorous methodology that they usually follow.^20^ ^,^ ^21^ We have not excluded systematic reviews from other specific journals, namely Campbell Systematic Reviews and JBI journals.

#### 2.3.2. Sampling and study size

We retrieved a random sample of systematic reviews stratified by the JCR category (Supplementary Table 1). For each category, we applied the query TS = (‘systematic review’)—along with the identification of the respective category and with the 2010–2020 publication year filter—to search the Web of Science Core Collection (date last searched: 03.01.2022). We selected those systematic reviews whose sorting number (with results having been sorted by date in Web of Science) was listed in a randomly generated set of pre-selected numbers. We included a similar number of systematic reviews of each category in our study. The primary outcome of this assessment consisted of the proportion of each item fulfilled in our tool. We estimated that we would need to assess at least 385 systematic reviews to ensure a maximum margin of error of 5 percent points in 95% confidence intervals for those proportions, assuming a ‘population’ of 112,498 articles claiming to be ‘systematic reviews’ and published between 2010 and 2020. We assumed a proportion of 50% of systematic reviews positively fulfilling each item (the most conservative estimate for proportions). We considered the standard error for proportions ( 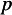 ) to be estimated by 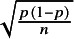 , with 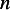 corresponding to the sample size.

#### 2.3.3 Variables

For each systematic review included in this study, we collected information (from the Web of Science platform) on the publication year, number of authors, number of references cited, region of publication, medical JCR categories, JIF, and percentile of JIF (JIF and percentile of JIF were retrieved on the year of publication; for systematic reviews with multiple JCR categories, for the percentile of JIF, we considered the highest percentile among the categories).

The region of publication was defined based on the country of the corresponding author’s address. We considered six regions of publication: Africa and Western and Southern Asia (thus, combining the African continent and Middle East and North Africa), Oceania, Eastern and Southeastern Asia, Europe, North America, and South America. This allowed us to follow a division through continents, except—for reasons grounded on the number of publications in our sample—the Asiatic and African regions. In fact, we opted to combine the African region with the Middle East, extending the MENA region^22^ and adjusting the Asiatic region accordingly.

We considered 38 Medical JCR categories (Supplementary Table 1). For purposes of this analysis, and in order to reduce the number of individual categories, we applied a non-supervised hierarchical clustering algorithm (complete-linkage clustering) after collecting our sample. This algorithm creates groups based on dissimilar distances between observations. Each of the included systematic reviews was an observation and JCR categories were included as features for the clustering model. The algorithm therefore identified categories that frequently appeared together in multiple reviews as more similar (i.e., having less dissimilarity) compared to those that seldom appeared together. This method provides a step-by-step splitting, indicating that categories in the first split (group A) are progressively more dissimilar to those in subsequent splits, with the last split (group G) being the most dissimilar. As a result, article categories ended up being classed into seven groups (listed in Supplementary Table 1). This algorithm allowed for each systematic review to be ascribed to one group only. If a systematic review had multiple categories that did not fall within the same group, it would be included in the first group that contained one of those categories, going from group A to group G, until a matching category was found, following the order of dissimilarity between the groups.

### 2.4 Data analysis

**Figure 1:**
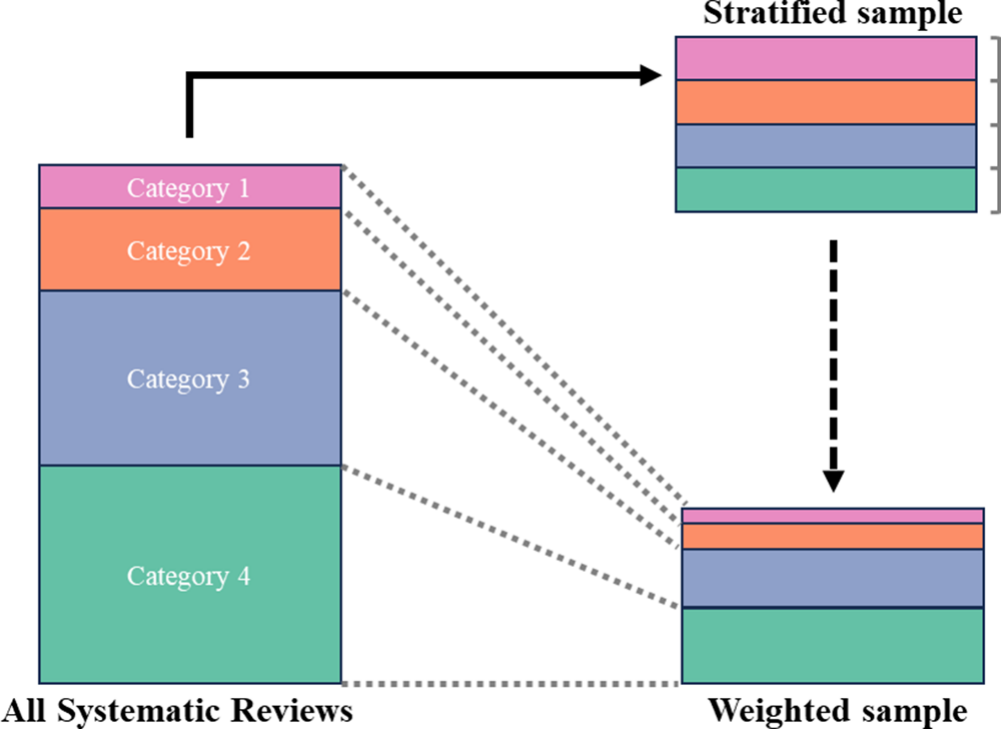
Graphical representation of the selection process of systematic reviews according to their categories into stratified (main analysis) and weighted samples.

The characteristics of the systematic reviews were separately described for systematic reviews with and without meta-analysis. Additionally, we computed the proportion of each ReMarQ item fulfilled. In order to obtain estimates of the proportions of fulfilled items generalisable for all systematic reviews, and overcoming the fact that we assessed a stratified sample with the same number of articles per category, we performed a sensitivity analysis in which we computed the proportions of each fulfilled item weighting for the frequency of published systematic reviews by JCR category (i.e., categories with larger numbers of systematic reviews would ‘weigh’ more than categories with a smaller number). Figure 1 schematically illustrates the process resulting in this weighted estimation. Systematic reviews with multiple JCR categories were weighted according to a single category, specifically the category for which their percentile of JIF was the highest.

Categorical variables were described with absolute and relative (%) frequencies and continuous variables were described with medians and interquartile ranges (IQR).

We built univariable and multivariable quasi-Poisson regression models to assess factors potentially associated with the reported methodological quality of systematic reviews. The outcome variable consisted of the number of ReMarQ items fulfilled among those 20 assessing the methodology of systematic reviews (i.e., those quality items which did not concern meta-analysis). The independent variables tested were the publication year, number of references cited, number of authors, JIF, percentile of JIF, whether meta-analysis was performed, category group, and region of publication. Exponentials of coefficients were interpreted as rate ratios (RR).

Data analysis was performed using R version 4.0.2^23^ and packages caret, ggmirt, MASS, mirt, rpart, and rpart.plot.^24^ ^–^ ^29^ 95% confidence intervals (CI) for point estimates were calculated. *p* Values < 0.05 were considered statistically significant.

## 3 Results

### 3.1. Descriptive analysis

We assessed a total of 400 systematic reviews, including 196 (49.0%) with meta-analysis. The characteristics of the assessed systematic reviews are presented in Table 1.

**Table 1:** Characteristics of assessed systematic reviews

	Total	SR only	SR with MA	
	(*N* = 400)	(*N* = 204)	(*N* = 196)	*p* Value
Quartiles of impact factor—*N (%)*				
Quartile 1	186 (46.5)	95 (46.6)	91 (46.4)	1.000*
Quartile 2	117 (29.3)	57 (27.9)	60 (30.6)	0.633*
Quartile 3	72 (18.0)	37 (18.1)	35 (17.9)	1.000*
Quartile 4	25 (6.3)	15 (7.4)	10 (5.1)	0.470*
Percentile of impact factor—*median (IQR)*	72.2 (51.5–90.2)	72.2 (49.8–91.4)	72.2 (53.4–89.4)	0.924*
Publication year—*N (%)*				
2010–2015	138 (34.5)	77 (37.7)	61 (31.1)	0.198*
2016–2020	262 (65.5)	127 (62.3)	135 (68.9)	
Journal impact factor—*median (IQR)*	3.0 (2.0–4.4)	2.8 (1.9–4.3)	3.2 (2.1–4.5)	0.120**
Number of authors—*median (IQR)*	5 (4–7)	5 (3–7)	6 (4–7)	<0.001**
Number of cited references—*median (IQR)*	47 (35–65)	49 (35–72)	45 (34–59)	0.031**
Journal categories—*median* (IQR)	2 (1–2)	2 (1–3)	2 (1–2)	0.042**
Group of categories a—*N (%)*				
Group A	72 (18.0)	37 (18.1)	35 (17.9)	1.000*
Group B	47 (11.8)	15 (7.4)	32 (16.3)	0.009*
Group C	32 (8.0)	16 (7.8)	16 (8.2)	1.000*
Group D	23 (5.8)	8 (3.9)	15 (7.7)	0.165*
Group E	16 (4.0)	11 (5.4)	5 (2.6)	0.232*
Group F	24 (6.0)	13 (6.4)	11 (5.6)	0.913*
Group G	186 (46.5)	104 (51.0)	82 (41.8)	0.083*
Region of publication—*N (%)*				
Africa, Western and Southern Asia	34 (8.5)	13 (6.4)	21 (10.3)	0.168*
Oceania	37 (9.3)	21 (1.3)	16 (8.2)	0.574*
Eastern and Southeastern Asia	48 (12.0)	8 (3.9)	40 (20.4)	<0.001*
Europe	164 (41.0)	104 (51.0)	60 (30.6)	<0.001*
North America	96 (24.0)	53 (26.0)	43 (21.9)	0.407*
South America	21 (5.3)	5 (2.5)	16 (8.2)	0.019*
SR ReMarQ items	10 (7–12)	8 (6–11)	11 (9–14)	<0.001**
fulfilled—median *(IQR)*				
MA ReMarQ items	*NA*	*NA*	4 (3–5)	
fulfilled—*median (IQR)*				
0 criteria—*N (%)*	*NA*	*NA*	3 (1.5)	
1 criterion*—N (%)*	*NA*	*NA*	6 (3.1)	
2 criteria*—N (%)*	*NA*	*NA*	20 (10.2)	
3 criteria*—N (%)*	*NA*	*NA*	60 (30.6)	
4 criteria*—N (%)*	*NA*	*NA*	38 (19.4)	
5 criteria*—N (%)*	*NA*	*NA*	49 (25.0)	
6 criteria—*N (%)*	*NA*	*NA*	20 (10.2)	

*Note*: NA, not applicable; IQR, interquartile range; SD, standard deviation; *Chi-square test, **Mann–Whitney *U* test; *p*-value refers to the comparison between systematic reviews with and without meta-analysis. Group A encompasses Critical Care Medicine, Emergency Medicine, Orthopedics and Surgery; Group B encompasses Cardiac & Cardiovascular Systems, Hematology, Peripheral Vascular Disease and Respiratory System; Group C encompasses Oncology and Pharmacology & Pharmacy; Group D encompasses Genetics & Heredity and Medicine, Research & Experimental; Group E encompasses Pediatrics; Group F encompasses Clinical Neurology, Neuroimaging and Radiology, Nuclear Medicine & Medical Imaging; Group G encompasses Allergy, Anesthesiology, Dentistry, Oral Surgery & Medicine, Dermatology, Endocrinology & Metabolism, Gastroenterology & Hepatology, Gerontology, Infectious Diseases, Medical Informatics, Medicine, General & Internal, Medicine, Legal, Obstetrics & Gynecology, Ophthalmology, Otorhinolaryngology, Pathology, Primary Health Care, Psychiatry, Public, Environmental & Occupational Health, Rheumatology, Transplantation, Tropical Medicine, Urology & Nephrology.

Almost half of the assessed systematic reviews were published in journals from the first quartile of JIF (46.5%), with the median JIF being of 3.0. Almost two thirds (65.0%) of the assessed systematic reviews had European (41.0%) or North American (24.0%) researchers as corresponding authors. The median number of ReMarQ items fulfilled was 10 (IQR: 7–12). For the meta-analysis component (six quality items), a median of 4 items fulfilled was observed (IQR: 3–5). There were no statistically significant differences between systematic reviews with or without meta-analysis regarding the JIF (*p* = 0.120) or the percentile of the JIF (*p* = 0.924).

**Figure 2:**
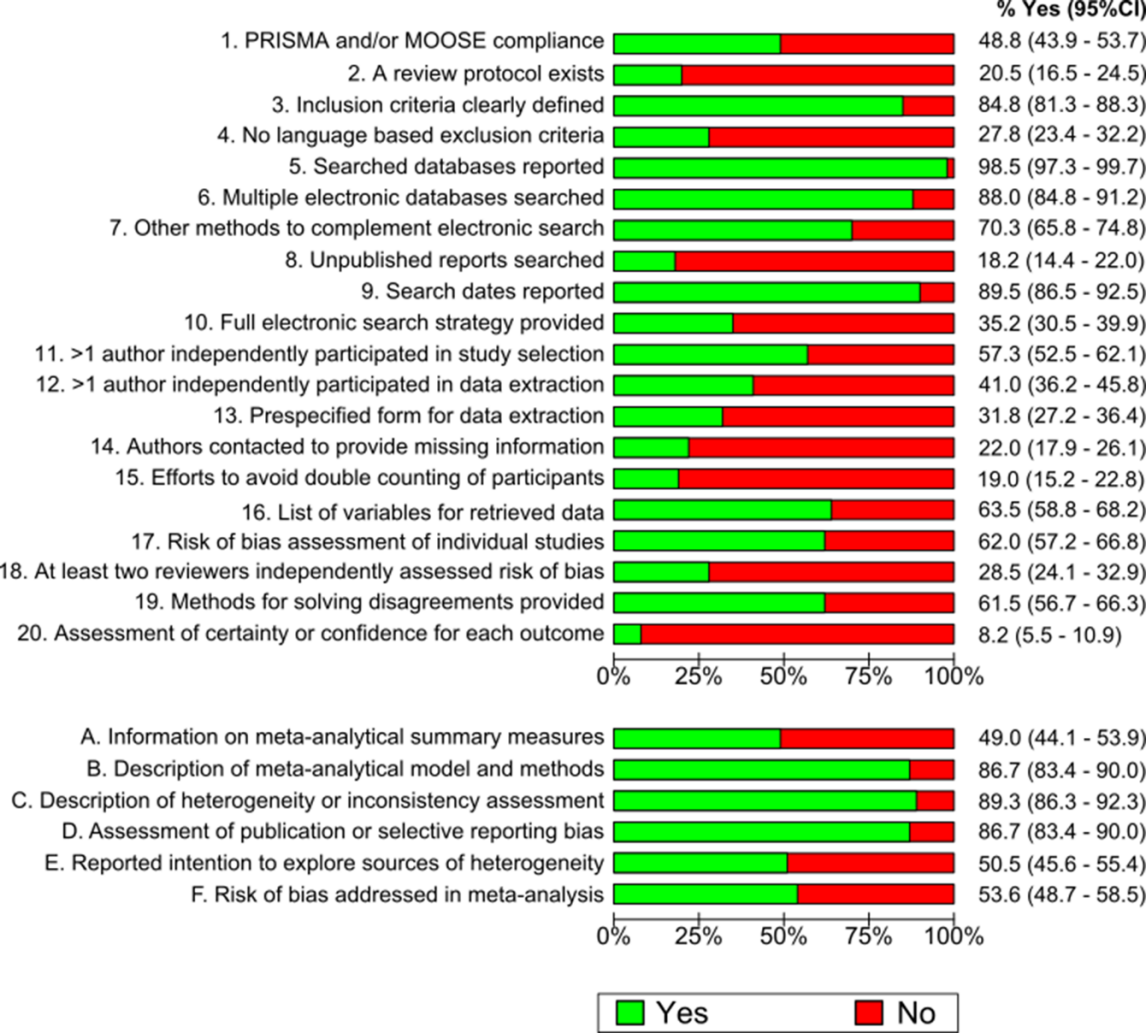
Distribution of fulfilled ReMarQ items (% Yes) of all systematic reviews (A) and of meta-analyses (B) Includes 400 systematic reviews stratified by JCR category (*n* = 38), where each category contributes with a similar number of systematic reviews (main analysis).

**Figure 3:**
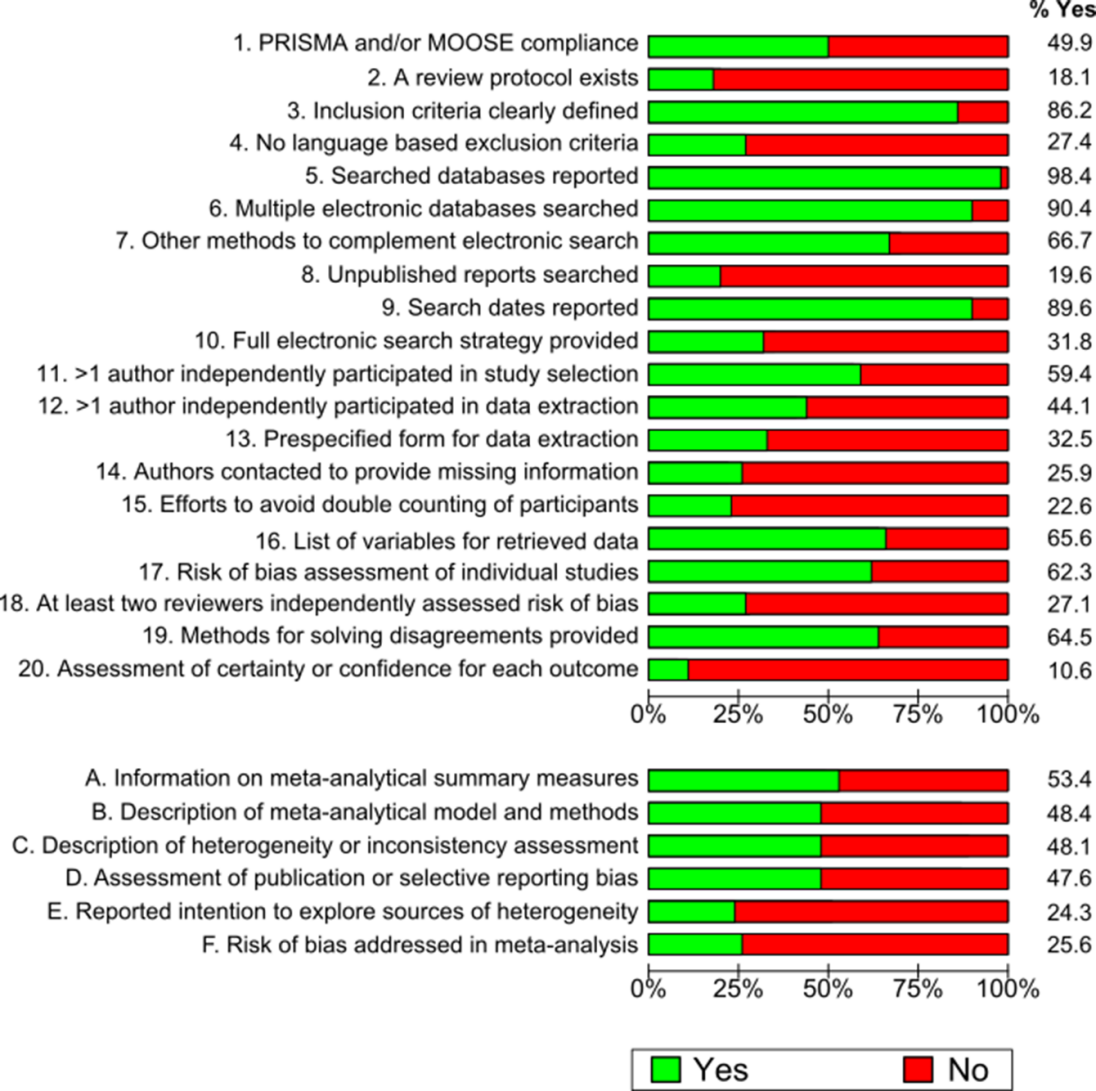
Distribution of ReMarQ fulfilled items (% Yes) of all systematic reviews (A) and of meta-analyses (B) weighted for the frequency of published systematic reviews by medical Journal Citation Reports category. Analysis corrected for the proportion of JCR category considering all systematic reviews published between 2010 and 2020 (sensitivity analysis).

Figure 2 shows the proportional distribution of fulfilled items in ReMarQ. The most frequently fulfilled items concerned the reporting of the search dates (90.0%; 95% CI = 87.1–92.9%) or of the searched bibliographic sources (98.0%; 95% CI = 96.6–99.4%), and indicating that multiple electronic bibliographic databases were searched (88.0%; 95% CI = 84.8–91.2%). By contrast, the least frequently fulfilled items concerned the assessment of certainty in the body of evidence (8.0%; 95% CI = 5.3–10.7%), the explicit search for information from unpublished sources (18.0%; 95% CI = 14.2–21.8%) and the availability of a review protocol and of its information (20.0%; 95% CI = 16.1–23.9%). Regarding systematic reviews with meta-analysis, 87.0% (95% CI = 83.7–90.3%) provided information on meta-analytical summary measures, 89.0% (95% CI = 85.9–92.1%) described the applied meta-analytical model and methods and 87.0% (95% CI = 83.7–90.3%) described heterogeneity or inconsistency assessment methods. Figure 3 shows the results of the sensitivity analysis weighted for the number of systematic reviews published by category of the JCR. The proportion of ReMarQ items fulfilled was similar when presenting weighted and unweighted results. The only exception concerned the ReMarQ items on meta-analysis, for which the proportion of fulfilled items tended to be lower when providing weighted results.

### 3.2 Properties of ReMarQ

#### 3.2.1. Difficulty and discrimination of items

Table 2 shows the item difficulty and discrimination for ReMarQ based on the IRT two-parameter logistic model, both for all systematic reviews and for systematic reviews with meta-analysis.

**Table 2:** Item difficulty and discrimination for ReMarQ assessed based on an item response theory two-parameter logistic model

	Systematic reviews (*n* = 400)		Systematic reviews with meta-analysis (*n* = 196)	
ReMarQ items	Difficulty	Discrimination	Difficulty	Discrimination
	(95% CI)	(95% CI)	(95% CI)	(95% CI)
Q1. PRISMA and/or MOOSE compliance.	0.07 (−0.18 to 0.31)	0.96 (0.65 to 1.26)	−0.66 (−1.17 to −0.15)	0.78 (0.35 to 1.21)
Q2. A review protocol exists.	1.22 (0.92 to 1.52)	1.57 (1.07 to 2.08)	0.98 (0.56 to 1.39)	1.36 (0.71 to 2.02)
Q3. Inclusion criteria are clearly defined.	−1.73 (−2.20 to −1.27)	1.26 (0.82 to 1.70)	−3.35 (−5.53 to −1.18)	0.89 (0.20 to 1.57)
Q4. No language-based exclusion criteria.	1.62 (0.91 to 2.34)	0.64 (0.35 to 0.93)	0.82 (0.27 to 1.38)	0.78 (0.35 to 1.21)
Q5. Searched databases reported.	−4.65 (−8.18 to −1.13)	1.01 (0.07 to 1.94)	−3.16 (−4.88 to −1.45)	1.80 (0.15 to 3.45)
Q6. Multiple electronic databases were searched.	−2.21 (−2.91 to −1.51)	1.09 (0.65 to 1.52)	−2.57 (−3.82 to −1.32)	1.23 (0.42 to 2.04)
Q7. Other methods to complement electronic search.	−2.08 (−3.34 to −0.83)	0.43 (0.17 to 0.69)	−3.18 (−6.93 to 0.58)	0.31 (−0.06 to 0.68)
Q8. Unpublished reports searched.	1.89 (1.24 to 2.54)	0.92 (0.55 to 1.29)	1.44 (0.78 to 2.10)	1.04 (0.49 to 1.59)
Q9. Searched dates reported.	−2.95 (−4.21 to −1.70)	0.81 (0.41 to 1.21)	−11.27 (−40.94 to 18.40)	0.24 (−0.40 to 0.87)
Q10. Full electronic search strategy provided.	0.91 (0.50 to 1.32)	0.76 (0.47 to 1.05)	1.00 (0.35 to 1.64)	0.74 (0.30 to 1.12)
Q11. >1 author independently participated in the study selection.	−0.29 (−0.49 to −0.08)	1.31 (0.94 to 1.69)	−0.84 (−1.37 to −0.31)	0.83 (0.40 to 1.27)
Q12. >1 author independently participated in data extraction.	0.42 (0.18 to 0.66)	1.09 (0.75 to 1.43)	−0.29 (−0.75 to 0.18)	0.71 (0.30 to 1.12)
Q13. Pre-specified form for data extraction.	0.98 (0.62 to 1.35)	0.92 (0.60 to 1.24)	0.73 (0.13 to 1.33)	0.66 (0.26 to 1.06)
Q14. Authors contacted to provide the missing information.	1.24 (0.91 to 1.57)	1.35 (0.91 to 1.79)	0.77 (0.31 to 1.24)	0.94 (0.47 to 1.40)
Q15. Efforts to avoid double counting of participants.	3.88 (0.99 to 6.77)	0.39 (0.09 to 0.68)	4.46 (−3.98 to 12.91)	0.19 (−0.17 to 0.55)
Q16. List of variables for retrieved data.	−0.75 (−1.10 to −0.41)	0.84 (0.55 to 1.14)	−3.22 (−6.57 to 0.13)	0.37 (−0.02 to 0.76)
Q17. Risk of bias assessment of individual studies.	−0.40 (−0.57 to −0.22)	1.91 (1.36 to 2.46)	−0.90 (−1.19 to −0.60)	2.45 (1.18 to 3.72)
Q18. At least two reviewers independently assessed the risk of bias.	0.80 (0.58 to 1.02)	1.74 (1.21 to 2.27)	0.44 (0.16 to 0.72)	1.59 (0.89 to 2.29)
Q19. Methods for solving disagreements are provided.	−0.34 (−0.50 to −0.18)	2.38 (1.66 to 3.10)	−0.99 (−1.40 to −0.59)	1.42 (0.77 to 2.06)
Q20. Assessment of certainty or confidence for each outcome.	2.00 (1.48 to 2.52)	1.68 (0.99 to 2.37)	1.94 (1.10 to 2.79)	1.20 (0.51 to 1.89)
A. Information on meta-analytical summary measures.	NA	NA	−2.46 (−3.81 to −1.11)	0.87 (0.31 to 1.42)
B. Description of meta-analytical models and methods.	NA	NA	−3.07 (−5.12 to −1.03)	0.76 (0.18 to 1.34)
C. Description of heterogeneity or inconsistency assessment.	NA	NA	−2.75 (−4.47 to −1.04)	0.75 (0.22 to 1.29)
D. Assessment of publication or selective reporting bias.	NA	NA	−0.03 (−0.44 to 0.39)	0.77 (0.34 to 1.20)
E. Reported intention to explore sources of heterogeneity.	NA	NA	−0.27 (−0.83 to 0.29)	0.57 (0.18 to 0.95)
F. Risk of bias addressed in meta-analysis.	NA	NA	2.02 (1.11 to 2.93)	1.21 (0.48 to 1.94)

*Note*: NA, not applicable. Higher values of difficulty (red and orange tones) represent ReMarQ items that are harder to fulfil, while lower values (yellow and green tones) are easier to fulfil. The value represents the normalized classification obtained on the tool where a systematic review has a 50% chance of meeting the criteria. Lower values of discrimination (red and orange tones) represent ReMarQ items that have a lower ability to differentiate between systematic reviews of higher *versus* lower overall quality, while higher values (yellow and green tones) are better at distinguishing systematic reviews.

The item displaying the lowest difficulty (i.e., with the highest likelihood of being fulfilled) was the reporting of searched databases (Q5) (coefficient = −4.65; 95% CI = −8.18; −1.13). The one displaying the highest difficulty was the reporting of efforts to avoid the double counting of participants (Q15) (coefficient = 3.88; 95% CI = 0.99; 6.77). Risk of bias assessment of individual studies (Q17) and reporting of methods for solving disagreements (Q19) were the most discriminative items, with a discrimination of 1.91 (95% CI = 1.36; 2.46) and 2.38 (95% CI = 1.66; 3.10), respectively. Consistent results were observed for systematic reviews with meta-analysis, although with additional questions having been identified as of low difficulty.

### 3.3 Identification of items capable of predicting the reported methodological quality of systematic reviews

**Figure 4:**
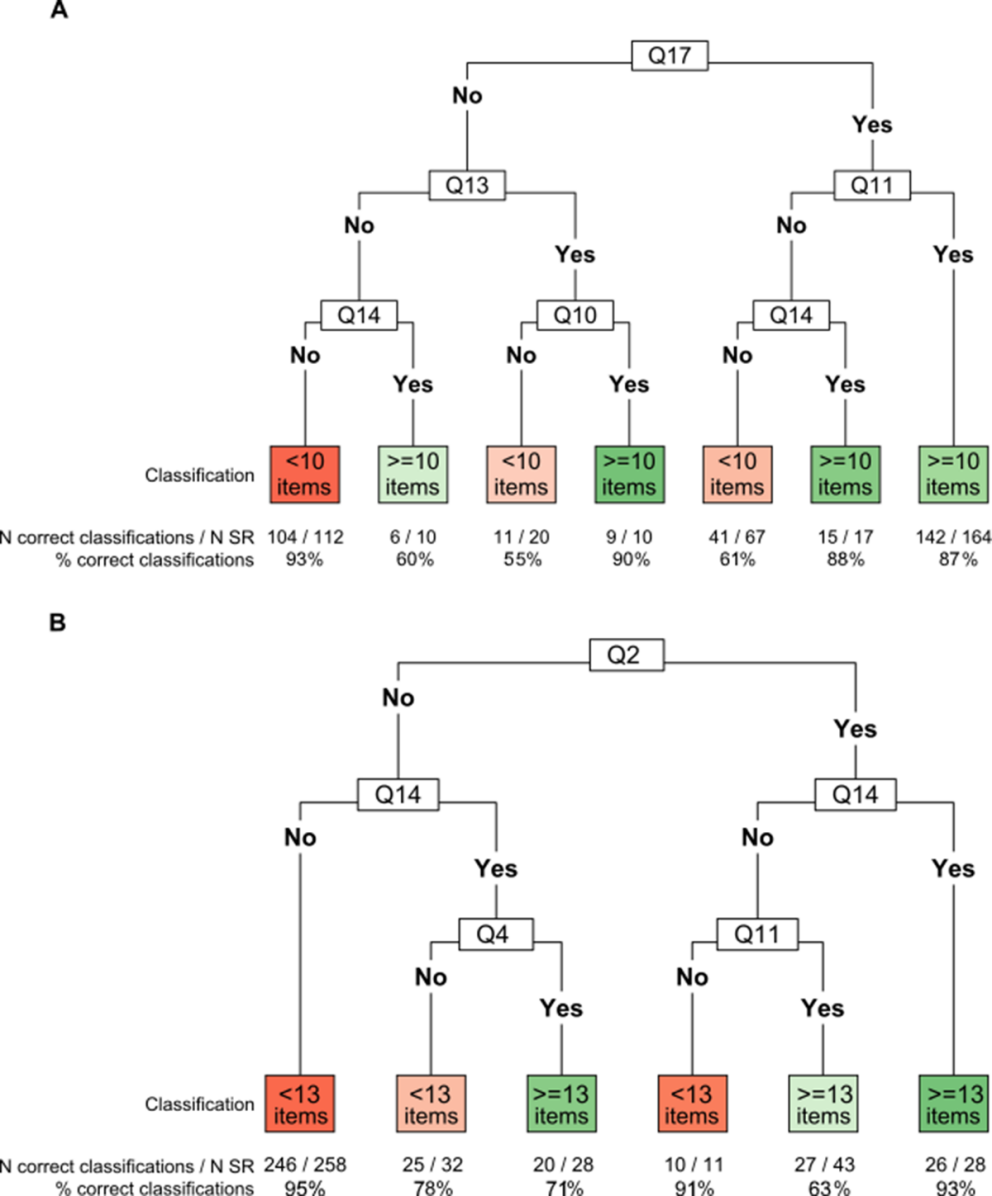
Classification trees to assess at least half (10 items or more) (A) and at least two thirds (13 items or more) (B) of ReMarQ items fulfilled. Q, quality item; SR, systematic review; Q2. A review protocol exists and its registration information was available. Q4. No language-based exclusion criteria were defined. Q10. The full electronic search strategy was provided for at least one database. Q11. Efforts were made to minimise error in the selection of studies, namely by having more than one author independently participating in the study selection process. Q13. Efforts were made to minimise error in data collection by using a prespecified form for data extraction from reports. Q14. Processes for obtaining and confirming data from investigators were described. Q17. The risk of bias (or methodological quality) of individual studies was formally assessed using appropriate criteria.

Figure 4 depicts the classification trees aiming to identify the sets of ReMarQ items that would more accurately predict that (A) at least half and (B) at least two thirds of quality items would be fulfilled.

Regarding the model concerning the median number of quality items, the accuracy of the classification tree was 81.5% (95% CI = 77.3–85.2%) and the kappa coefficient was 0.63 (95% CI = 0.53–0.73), attaining a sensitivity of 86.9% (95% CI = 81.3–91.3%), a specificity of 76.6% (95% CI = 70.2–82.1%), a positive predictive value (PPV) of 77.2% (95% CI = 71.0–82.6%) and a negative predictive value (NPV) of 86.5% (95% CI = 80.7–91.1%). The first node quality item (i.e., the most informative quality item for assessment of the overall ReMarQ score) corresponded to that assessing whether a risk of bias assessment of individual studies was conducted (Q17). We observed that a systematic review with (i) no risk of bias assessment (Q17), (ii) no reporting of use of a prespecified form for data extraction (Q13) and (iii) no reporting of contact with the study’s authors to obtain and/or confirm data (Q14) had a probability of 92.9% (95% CI = 86.4–96.9%) of having less than the median number of fulfilled ReMarQ items. Conversely, a systematic review with risk of bias assessment (Q17) describing that more than one author independently participated in the study selection process (Q11) displayed an 86.6% (95% CI = 80.4–91.4%) probability of having more than the median number of fulfilled ReMarQ items.

When considering the occurrence of two thirds of ReMarQ items fulfilled, the classification tree attained an accuracy of 88.5% (95% CI = 85.0–91.5%) and a kappa coefficient of 0.68 (95% CI = 0.59–0.78). Sensitivity was 73.7% (95% CI = 63.9–82.1%), specificity was 93.4% (95% CI = 89.9–95.9%), PPV 78.5% (95% CI = 68.8–86.3%) and NPV 91.5% (95% CI = 87.8–94.4%). The first node quality item corresponded to that assessing whether a review protocol was available (Q2). We observed that a systematic review with no available review protocol (Q2) and no reporting of contact with the study’s authors to obtain and/or confirm data (Q14) had a probability of 95.4% (95% CI = 92.0–97.6%) of having less than two thirds of ReMarQ items fulfilled. Similarly, a systematic review with an available review protocol (Q2) and referring to the fact that investigators were contacted (Q14) displayed a 92.9% (95% CI = 76.5–99.1%) probability of having more than two thirds of ReMarQ items fulfilled.

### 3.4 Assessment of differences between PRISMA 2020 Checklist items and ReMarQ

The PRISMA 2020 Checklist^10^ was applied to a randomly selected subset of 100 systematic reviews obtained from the systematic reviews in our main analysis sample. Results of the fulfilment of PRISMA 2020 items were compared with results from ReMarQ (Supplementary File 1). For several aspects, namely those concerning the eligibility criteria, selection process, data collection process, risk of bias assessment, and synthesis methods (topics in the PRISMA 2020 checklist^10^), we found differences of at least 5 percent points when comparing the percentage of fulfilled items in ReMarQ versus those of PRISMA 2020. The highest discrepancy was observed for the item ‘specify the inclusion and exclusion criteria for the review and how studies were grouped for the syntheses’ (item #5^10^), which corresponded to the items ‘inclusion criteria for primary studies were clearly defined’ (Q3) and ‘no language-based exclusion criteria were defined’ (Q4) of our tool. In this item, 85.0% of systematic reviews were considered as fulfilling the PRISMA item, compared with an average of 58.5% for the corresponding items from our reported methodological quality tool. These differences point to the discriminant validity of ReMarQ compared to PRISMA.

### 3.5 Identification of factors associated with reported methodological quality

Table 3 shows the results of the univariable and multivariable quasi-Poisson regression models identifying factors associated with systematic reviews’ reported methodological quality. More recent systematic reviews were significantly associated with higher quality (i.e., with a higher number of fulfilled ReMarQ items) (adjusted RR = 1.03; 95% CI = 1.02–1.04, *p* < 0.001). Systematic reviews with meta-analysis were also associated with higher quality (adjusted RR = 1.34; 95% CI = 1.25–1.43, *p* < 0.001) than those without (this comparison considers only the items of the systematic review component of ReMarQ, for which a median of 11 was fulfilled for systematic reviews with meta-analysis versus 8 for systematic reviews without meta-analysis). The number of authors and the JIF did not reach statistical significance in the multivariable model, in contrast to univariable analyses. The JIF percentile did not associate with higher reported methodological quality, neither in the univariable nor in the multivariable models. Compared to systematic reviews published in North America, those published in Oceania, Eastern and Southern Asia, and South America tended to display a better methodological quality reported. However, these differences mostly ceased to be observed in the multivariable models.

**Table 3:** Results of the univariable and multivariable models identifying factors associated with the reported methodological quality of systematic reviews

	Univariable model RR (95% CI) [*p*-value]	Multivariable model RR (95% CI) [*p*-value]
Publication year	1.03 (1.02 to 1.05) [<0.001]	1.03 (1.02 to 1.04) [<0.001]
References cited^a^	0.98 (0.97 to 0.99) [0.009]	0.99 (0.97 to 1.00) [0.026]
Number of authors	1.02 (1.01 to 1.03) [<0.001]	1.01 (1.00 to 1.02) [0.038]
Journal impact factor	1.01 (1.00 to 1.02) [0.029]	1.00 (0.99 to 1.01) [0.684]
Percentile of impact factor	1.00 (1.00 to 1.00) [0.407]	1.00 (1.00 to 1.00) [0.245]
Performed meta-analysis	1.38 (1.29 to 1.47) [<0.001]	1.34 (1.25 to 1.43) [<0.001]
Group of categories^b^		
Group A	(*reference*)	(*reference*)
Group B	1.05 (0.93 to 1.20) [0.413]	0.99 (0.88 to 1.11) [0.819]
Group C	0.83 (0.71 to 0.97) [0.019]	0.86 (0.75 to 0.99) [0.041]
Group D	0.91 (0.76 to 1.08) [0.270]	0.88 (0.75 to 1.02) [0.097]
Group E	0.92 (0.75 to 1.12) [0.399]	0.99 (0.83 to 1.18) [0.932]
Group F	0.78 (0.65 to 0.93) [0.006]	0.78 (0.67 to 0.92) [0.003]
Group G	0.94 (0.85 to 1.04) [0.224]	0.95 (0.87 to 1.03) [0.237]
Region of publication		
North America	(*reference*)	(*reference*)
Africa, Western and Southern Asia	0.94 (0.81 to 1.09) [0.424]	0.89 (0.78 to 1.02) [0.088]
Oceania	1.14 (0.99 to 1.30) [0.064]	1.11 (0.98 to 1.25) [0.103]
Eastern and Southeastern Asia	1.14 (1.01 to 1.29) [0.038]	0.98 (0.87 to 1.09) [0.670]
Europe	1.01 (0.92 to 1.10) [0.897]	1.02 (0.94 to 1.11) [0.631]
South America	1.27 (1.09 to 1.49) [0.003]	1.16 (1.00 to 1.34) [0.052]

*Note:* RR, rate ratio. Group B encompasses Cardiac & Cardiovascular Systems, Hematology, Peripheral Vascular Disease and Respiratory System; Group C encompasses Oncology and Pharmacology & Pharmacy; Group D encompasses Genetics & Heredity and Medicine, Research & Experimental; Group E encompasses Pediatrics; Group F encompasses Clinical Neurology, Neuroimaging and Radiology, Nuclear Medicine & Medical Imaging; Group G encompasses Allergy, Anesthesiology, Dentistry, Oral Surgery & Medicine, Dermatology, Endocrinology & Metabolism, Gastroenterology & Hepatology, Gerontology, Infectious Diseases, Medical Informatics, Medicine, General & Internal, Medicine, Legal, Obstetrics & Gynecology, Ophthalmology, Otorhinolaryngology, Pathology, Primary Health Care, Psychiatry, Public, Environmental & Occupational Health, Rheumatology, Transplantation, Tropical Medicine, Urology & Nephrology.

a RR per each 10 references cited.

b Group A encompasses Critical Care Medicine, Emergency Medicine, Orthopedics and Surgery.

## 4 Discussion

In this study, we developed and tested the psychometric properties of ReMarQ - a tool for the assessment of the reported methodological quality of systematic reviews. Through an IRT model, we found that the items ‘risk of bias assessment of individual studies’ (Q17) and ‘reporting of methods for solving disagreements’ (Q19) were the most discriminant quality items between systematic reviews (i.e., the items most able to differentiate between systematic reviews according to their reported methodological quality). In classification trees, the assessment of the risk of bias of individual studies using appropriate criteria (Q17) was also identified as the most informative item for determining whether more than half of ReMarQ items were fulfilled, while the availability of a research protocol (Q2) was the most informative item for determining whether more than two thirds of ReMarQ items were fulfilled. In other words, these items were the most important predictors of whether a systematic review would fulfil at least half or two thirds of ReMarQ items, respectively. After applying ReMarQ to the Methods section of a random sample of medical systematic reviews, we identified a particularly low frequency of fulfilling several items, including (i) the existence of a review protocol, (ii) the absence of language-based exclusion criteria, and (iii) assessment of the confidence in the obtained evidence. A suboptimal frequency of fulfilling was observed for other items such as the assessment of the risk of bias. Finally, we identified that a more recent publication date and the inclusion of a meta-analysis were associated with a higher reported methodological quality. For the publication date, even though the yearly effect size appears small (RR = 1.03), it corresponds to an adjusted RR of 1.34 for the whole period studied. Therefore, despite the massified increase in published systematic reviews,^3^ the overall reported methodological quality of systematic reviews appears to have significantly improved over the years. This observation may be, among others, an effect of the wider dissemination and adoption of the PRISMA checklist.^30^ ^,^ ^31^ However, the period covered in this study (2010–2020, decided to cover the period between the publication of the first PRISMA statement^15^ and the subsequent one^10^) does not fully reflect the changes in publication patterns associated with COVID-19. Consequently, the overall atypical reduction in publication quality that occurred primarily in COVID-19-related systematic reviews^32^ ^,^ ^33^ most likely did not influence our findings.

While the JIF is sometimes associated with the quality of publications,^34^ ^–^ ^36^ we found that neither the JIF nor the JIF percentile were associated with higher reported methodological quality. Our results may partly be due to the known right-skewness of the JIF (i.e., the JIF is influenced by a few highly cited papers).^37^ Nevertheless, and most importantly, our results underscore the argument that the JIF is a journal metric and should not be used for individual assessments of quality or to predict the future impact of publications.^35^ ^,^ ^38^ ^–^ ^41^ Therefore, a careful methodological assessment of systematic reviews is essential and irrespective of the journal in which they have been published or of the JIF.

For the reported methodological quality assessment of systematic reviews, we used a purposely built tool (ReMarQ), as this is a construct that it is not fully covered by the PRISMA statements^10^ ^,^ ^15^ or the ROBIS tool.^11^ Regarding PRISMA statements, we took into account questions in Section 2, as questions from other sections (i) may not have such a direct relationship with the methodological quality of the systematic review, since they are concerned with reporting transparency and completeness and (ii) may have a lower discriminative capacity. To test this claim, we applied the PRISMA 2020 Checklist^10^ to a subset of 100 systematic reviews and found differences in the proportion of fulfilled items between the PRISMA items and the items in ReMarQ. This further corroborates a need for a specific reported methodological quality tool. Regarding the ROBIS tool, by measuring the risk of bias construct, some of its questions may involve a certain degree of subjectiveness^11^ ^,^ ^13^ or need adequate specific clinical knowledge for correct implementation. Of note, these existing tools had been previously used by other authors to assess systematic reviews, although typically in more specific contexts (e.g., COVID-19).^32^ ^,^ ^33^ ^,^ ^42^ Given these more specific assessments, heterogeneous results were found—for example, there have been reports of very poor compliance with quality items when using a combination of the PRISMA Statement and the AMSTAR tool^43^ to systematic reviews in orthodontics.

This study has some limitations. We opted to exclude systematic reviews published in the Cochrane Database of Systematic Reviews journal (impairing the comparison between Cochrane and non-Cochrane systematic reviews), for which some authors deemed to uphold higher methodological rigour.^20^ ^,^ ^21^ This could have led to an underestimate of the proportion of fulfilled ReMarQ items in the whole spectrum of systematic reviews, with potential implications on the generalizability of our results. However, systematic reviews published in the Cochrane Database of Systematic Reviews represent only 0.5% of published systematic reviews during the considered period, meaning that end users are much more likely to encounter systematic reviews for which they are uncertain of the methodological quality. Regarding the systematic reviews selected, we retrieved a stratified sample according to the medical JCR categories. This meant that the distribution of categories in our sample did not correspond to that observed in the “population” of systematic reviews. Nonetheless, no major differences were found when performing a sensitivity analysis weighted by category. Additionally, considerations regarding some of the variables collected for each systematic review are necessary, namely for the number of references cited, the number of authors, the region of publication, and the publication date. The number of cited references and the number of authors have been shown to be associated with the number of citations an article will receive (a frequently used proxy measure for article quality).^34^ Regarding the former, several journals may limit the number of references an article can cite (which means that authors may not be totally responsible for the number of references in their systematic review). However, reviews are often exempted from such strict limits as those observed for other article types, and in our sample, no ‘ceiling effect’ on the number of references was observed. Regarding the region of publication, we used the corresponding author’s address and combined Africa with the Middle East. These are arguable options and may have influenced our analysis by potentially obscuring specific regional trends or biases. Furthermore, the merging of these regions reflects a compromise that may not fully account for the distinct scientific and cultural contributions of each area. However, this decision was driven by the need for a pragmatic analysis framework, given the limited number of studies from some regions and the overarching goal of our research to draw broader geographical comparisons. Lastly, the publication year corresponded to the official publication year, which in some cases may have happened a few weeks or months after any early online accesses specific to the journal where the systematic review was published. On the other hand, there may be a relevant time lag between the initiation of a systematic review and its publication year, given that the methodology can be time-consuming.^16^ In fact, a previous study has found that the time between protocol registration and publication is on average one and a half years.^44^

This study also has several strengths. The tool we present in this work (ReMarQ) has been developed based on a multistep process, starting from the consultation of the PRISMA statements,^10^ ^,^ ^15^ the ROBIS tool,^11^ and the Cochrane Handbook for Systematic Reviews.^16^ ReMarQ focuses on reported methodological quality, a construct for which an assessment tool was lacking. In addition, the nature of our tool—namely the fact that it encompasses dichotomous items and does not require previous clinical background on the subject being addressed for implementation—renders it as a potential basis for an automated tool supporting the assessment of the reported methodological quality of systematic reviews. In particular, a large language model could potentially be trained to apply the developed tool, providing a Yes/No answer for each of its items. Despite not being aimed at improving or controlling the quality of systematic reviews, automated processes could help in supporting a more efficient assessment of the methodological quality of systematic reviews. Analogous automated processes already exist for randomised controlled trials,^45^ but are yet to be developed for systematic reviews and meta-analyses.^46^ Lastly, the analytical methods we applied provide a description of the overall quality of systematic reviews and their association with the systematic reviews’ characteristics, providing relevant meta-research information.

In conclusion, we developed a tool capable of assessing the reported methodological quality of systematic reviews. We have evaluated its psychometric properties, identifying a relatively short set of quality items that were highly predictive of the overall reported methodological quality of systematic reviews. The low complexity of the items and the absence of needed subject content or methodologic expertise to apply our tool renders it possible to be easily applied by different end-users of systematic reviews. Additionally, due to the dichotomous nature of the items of the developed tool, ReMarQ can be the basis for studies aimed at the development of automated processes and tools for supporting a more efficient assessment of reported methodological quality of systematic reviews.

### 4.1. Potential impact

This study underscores the usefulness of ReMarQ to assist healthcare professionals, researchers, and decision-makers in the evaluation of the reported methodological quality of systematic reviews. We provide a tool that may help streamline the process of quality assessment of systematic reviews by their end-users given (i) its relatively low complexity of implementation and lack of need of content expertise and (ii) the dichotomous nature of its question, facilitating the development of automated processes based on ReMarQ.

## Supporting information

Marques-Cruz et al. supplementary material 1 Marques-Cruz et al. supplementary material

Marques-Cruz et al. supplementary material 2 Marques-Cruz et al. supplementary material

## Data Availability

The data that support the findings of this study are available from the corresponding author upon reasonable request.

## Supplementary material

To view supplementary material for this article, please visit http://doi.org/10.1017/rsm.2024.14.
