# Supplementary material for: Reported methodological quality of medical systematic reviews: Development of an assessment tool (ReMarQ) and meta-research study
Source: Res Synth Methods. 2025 Mar 7;16(1):175–93. doi: 10.1017/rsm.2024.14 (PMC12621523; doi:10.1017/rsm.2024.14)
Supplement: Marques-Cruz et al. supplementary material 1 — Marques-Cruz et al. supplementary material [file S1759287924000140sup001.docx]

**Supplementary File 1. Analysis of PRISMA 2020 Checklist items *vis-à-vis* those of our Reported Methodological Quality tool**

**Methodological notes**

We randomly selected a subset of 100 systematic reviews (SR) form the sample of 400 included in the study. We applied the PRISMA 2020 Checklist [1], except for the items from the PRISMA 2020 for Abstracts checklist. A PRISMA item was considered “fulfilled” if all of its aspects had been reported in the systematic review; in case only part of its aspects had been reported, the item was considered “partially fulfilled”.

We described the proportion of fulfilled items and compared these results with those obtained with our Reported Methodological Quality tool, by (i) comparing the overall and per section proportion of fulfilled PRISMA checklist items and (ii) by analyzing individual differences between the items from the Methods section that had some correspondence to items from the Reported Methodological Quality tool.

**I. Proportion of fulfilled items per topic**

Table 1 shows the percentage of fulfilled PRISMA 2020 Checklist items in the sample of 100 assessed systematic reviews. Partially fulfilled items represented instances where the item could not be considered either completely fulfilled or unfulfilled.

Six items were fulfilled in more than 90% of the times: “provide a general interpretation of the results in the context of other evidence” (23a), “provide an explicit statement of the objective(s) or question(s) the review addresses” (4), “describe the rationale for the review in the context of existing knowledge” (3), “discuss implications of the results for practice, policy, and future research” (23d), “cite each included study and present its characteristics” (17), and “specify all databases, registers, websites, organisations, reference lists and other sources searched or consulted to identify studies; specify the date when each source was last searched or consulted” (6).

Conversely, four items were fulfilled in less than 10% of times: “describe and explain any amendments to information provided at registration or in the protocol” (24c), “report which of the following are publicly available and where they can be found: template data collection forms; data extracted from included studies; data used for all analyses; analytic code; any other materials used in the review.” (27), “describe the processes used to decide which studies were eligible for each synthesis (e.g. tabulating the study intervention characteristics and comparing against the planned groups for each synthesis).” (13a), and “describe any methods used to assess certainty (or confidence) in the body of evidence for an outcome.” (15).

For four items, the proportions of systematic reviews in which such items were fulfilled or partially fulfilled differed by more than 5 percent points [pp]. In particular, this difference was of 5 pp in “describe any methods required to prepare the data for presentation or synthesis, such as handling of missing summary statistics, or data conversions” (13b); of 10 pp in “specify the methods used to decide whether a study met the inclusion criteria of the review, including how many reviewers screened each record and each report retrieved, whether they worked independently, and if applicable, details of automation tools used in the process” (8) and “specify the methods used to assess risk of bias in the included studies, including details of the tool(s) used, how many reviewers assessed each study and whether they worked independently, and if applicable, details of automation tools used in the process” (11); and 39 pp in “specify the methods used to collect data from reports, including how many reviewers collected data from each report, whether they worked independently, any processes for obtaining or confirming data from study investigators, and if applicable, details of automation tools used in the process.” (9).

**Table 1. Percentage of PRISMA 2020 items fulfilled.**

| **Section and Topic** | **Item #** | **Checklist item** | **Fulfilled (%)** | **At least partially fulfilled (%)** |
| --- | --- | --- | --- | --- |
| **TITLE** | | |  |  |
| Title | 1 | Identify the report as a systematic review. | 88 | 88 |
| **INTRODUCTION** | | |  |  |
| Rationale | 3 | Describe the rationale for the review in the context of existing knowledge. | 98 | 98 |
| Objectives | 4 | Provide an explicit statement of the objective(s) or question(s) the review addresses. | 99 | 99 |
| **METHODS** | | |  |  |
| Eligibility criteria | 5 | Specify the inclusion and exclusion criteria for the review and how studies were grouped for the syntheses. | 85 | 85 |
| Information sources | 6 | Specify all databases, registers, websites, organisations, reference lists and other sources searched or consulted to identify studies. Specify the date when each source was last searched or consulted. | 91 | 91 |
| Search strategy | 7 | Present the full search strategies for all databases, registers and websites, including any filters and limits used. | 42 | 42 |
| Selection process | 8 | Specify the methods used to decide whether a study met the inclusion criteria of the review, including how many reviewers screened each record and each report retrieved, whether they worked independently, and if applicable, details of automation tools used in the process. | 63 | 73 |
| Data collection process | 9 | Specify the methods used to collect data from reports, including how many reviewers collected data from each report, whether they worked independently, any processes for obtaining or confirming data from study investigators, and if applicable, details of automation tools used in the process. | 32 | 71 |
| Data items | 10a | List and define all outcomes for which data were sought. Specify whether all results that were compatible with each outcome domain in each study were sought (e.g. for all measures, time points, analyses), and if not, the methods used to decide which results to collect. | 63 | 63 |
|  | 10b | List and define all other variables for which data were sought (e.g. participant and intervention characteristics, funding sources). Describe any assumptions made about any missing or unclear information. | 63 | 63 |
| Study risk of bias assessment | 11 | Specify the methods used to assess risk of bias in the included studies, including details of the tool(s) used, how many reviewers assessed each study and whether they worked independently, and if applicable, details of automation tools used in the process. | 51 | 61 |
| Effect measures | 12 | Specify for each outcome the effect measure(s) (e.g. risk ratio, mean difference) used in the synthesis or presentation of results. | 38 | 38 |
| Synthesis methods | 13a | Describe the processes used to decide which studies were eligible for each synthesis (e.g. tabulating the study intervention characteristics and comparing against the planned groups for each synthesis (item #5)). | 9 | 9 |
|  | 13b | Describe any methods required to prepare the data for presentation or synthesis, such as handling of missing summary statistics, or data conversions. | 14 | 19 |
|  | 13c | Describe any methods used to tabulate or visually display results of individual studies and syntheses. | 18 | 18 |
|  | 13d | Describe any methods used to synthesize results and provide a rationale for the choice(s). If meta-analysis was performed, describe the model(s), method(s) to identify the presence and extent of statistical heterogeneity, and software package(s) used. | 35 | 37 |
|  | 13e | Describe any methods used to explore possible causes of heterogeneity among study results (e.g. subgroup analysis, meta-regression). | 19 | 19 |
|  | 13f | Describe any sensitivity analyses conducted to assess robustness of the synthesized results. | 19 | 19 |
| Reporting bias assessment | 14 | Describe any methods used to assess risk of bias due to missing results in a synthesis (arising from reporting biases). | 21 | 21 |
| Certainty assessment | 15 | Describe any methods used to assess certainty (or confidence) in the body of evidence for an outcome. | 9 | 9 |
| **RESULTS** | | |  |  |
| Study selection | 16a | Describe the results of the search and selection process, from the number of records identified in the search to the number of studies included in the review, ideally using a flow diagram. | 87 | 87 |
|  | 16b | Cite studies that might appear to meet the inclusion criteria, but which were excluded, and explain why they were excluded. | 22 | 26 |
| Study characteristics | 17 | Cite each included study and present its characteristics. | 92 | 92 |
| Risk of bias in studies | 18 | Present assessments of risk of bias for each included study. | 55 | 55 |
| Results of individual studies | 19 | For all outcomes, present, for each study: (a) summary statistics for each group (where appropriate) and (b) an effect estimate and its precision (e.g. confidence/credible interval), ideally using structured tables or plots. | 53 | 55 |
| Results of syntheses | 20a | For each synthesis, briefly summarise the characteristics and risk of bias among contributing studies. | 24 | 27 |
|  | 20b | Present results of all statistical syntheses conducted. If meta-analysis was done, present for each the summary estimate and its precision (e.g. confidence/credible interval) and measures of statistical heterogeneity. If comparing groups, describe the direction of the effect. | 46 | 46 |
|  | 20c | Present results of all investigations of possible causes of heterogeneity among study results. | 18 | 18 |
|  | 20d | Present results of all sensitivity analyses conducted to assess the robustness of the synthesized results. | 18 | 18 |
| Reporting biases | 21 | Present assessments of risk of bias due to missing results (arising from reporting biases) for each synthesis assessed. | 18 | 18 |
| Certainty of evidence | 22 | Present assessments of certainty (or confidence) in the body of evidence for each outcome assessed. | 33 | 33 |
| **DISCUSSION** | | |  |  |
| Discussion | 23a | Provide a general interpretation of the results in the context of other evidence. | 100 | 100 |
|  | 23b | Discuss any limitations of the evidence included in the review. | 81 | 81 |
|  | 23c | Discuss any limitations of the review processes used. | 57 | 57 |
|  | 23d | Discuss implications of the results for practice, policy, and future research. | 95 | 95 |
| **OTHER INFORMATION** | | |  |  |
| Registration and protocol | 24a | Provide registration information for the review, including register name and registration number, or state that the review was not registered. | 22 | 22 |
|  | 24b | Indicate where the review protocol can be accessed, or state that a protocol was not prepared. | 22 | 22 |
|  | 24c | Describe and explain any amendments to information provided at registration or in the protocol. | 1 | 1 |
| Support | 25 | Describe sources of financial or non-financial support for the review, and the role of the funders or sponsors in the review. | 66 | 66 |
| Competing interests | 26 | Declare any competing interests of review authors. | 86 | 86 |
| Availability of data, code and other materials | 27 | Report which of the following are publicly available and where they can be found: template data collection forms; data extracted from included studies; data used for all analyses; analytic code; any other materials used in the review. | 7 | 7 |

“%” represents the percentage of systematic reviews that fulfil a given item. 1% corresponds to one systematic review. “At least partially fulfilled” includes instances where the item could not be considered either completely fulfilled or unfulfilled.

**II. Comparison between fulfilled items in the PRISMA Checklist and in our Reported Methodological Quality tool**

Figure 1 shows the comparison between the number of items fulfilled in our Reported Methodological Quality tool and the proportion of completely fulfilled items in PRISMA 2020 Checklist (overall and per section). SR were divided in deciles according to the number of items fulfilled in our Reported Methodological Quality tool.

An increase in the number of fulfilled items in our Reported Methodological Quality tool was associated with an increase in the number of fulfilled PRISMA. In fact, while the first decile (D1) of systematic reviews (according to our Reported Methodological quality tool) displayed an average of 9.7 fulfilled PRISMA items, the tenth decile (D10) displayed an average of 28.4 fulfilled PRISMA items. However, the only drivers of that increase were the Methods and Results sections, for which there were average increases of 10.1 and 5.7 PRISMA fulfilled items, respectively. By contrast, we found no relevant changes in the number of items fulfilled in the Title, Introduction, Discussion and Other information sections of the PRISMA 2020 Checklist. This further corroborates our premise that the PRISMA Statement does not cover the “reported methodological quality” construct, but it rather concerns transparency and completeness of reporting.

**Figure 1. PRISMA 2020 Checklist and Section fulfilment according to deciles of items fulfilled in the Reported Methodological Quality tool.**

**
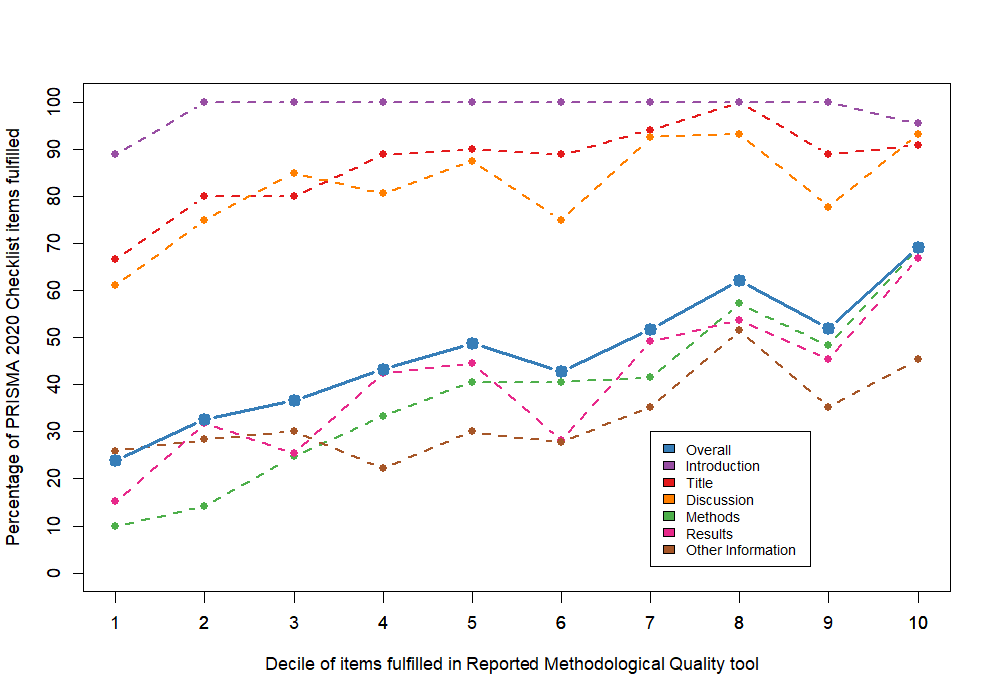
**

Note: Colours are presented in the legend from the most fulfilled section to the least, except for “Overall”. Each point represents the average proportion of items fulfilled in the PRISMA 2020 Checklist by the respective decile of items fulfilled in the Reported Methodological Quality tool. Deciles (D) were included the following range of items fulfilled: D1 = [2,5[; D2 = [5,7[; D3 = [7,8[; D4 = [8,9[; D5 = [9,10[; D6 = [10,11[; D7 = [11,13[; D8 = [13,13.2[; D9 = [13.2,15[; D10 = [15,20].

**III. Comparison between PRISMA Checklist fulfilled items and Reported Methodological Quality tool**

Our Reported Methodological Quality tool was built based on several items of the PRISMA Checklists [1,2]. In particular, we identified a set of statements pertaining reported methodological quality and developed items so that they corresponded to a set of dichotomous statements (“yes/no” statements, for which “yes” indicated that an item was fulfilled). Hence, several items of our tool can be mapped as having some correspondence with the PRISMA Checklist items (Supplementary Table 1). We analysed the items that could have correspondence between the two tools, with such mapping (along with the frequence of fulfilled items) being displayed in Table 2). We present aggregated results for cases where multiple items from one tool correspond to a single item in the other tool. For items A-F in our tool, we only analysed the fulfillment proportion in SR with meta-analysis (which corresponded to 41% of our subset) for both tools. Partially fulfilled items in PRISMA Checklist were considered to correspond to a 50% fulfillment on that specific item, regardless of the actual proportion of the item that could be considered fulfilled.

According to the topics in the PRISMA 2020 Checklist, differences on the assessments of the tools were found in:

- Eligibility criteria – 85.0% (PRISMA item #5) vs 58.5% (Q2-3); 26.5 pp difference
- Selection process – 68.0% (PRISMA item #8) vs 51.0% (Q11); 17.0 pp difference
- Data collection process – 51.5% (PRISMA item #9) vs 28.5% (Q12-15); 23.0 pp difference
- Study risk of bias assessment – 56.0% (PRISMA item #11) vs 50.0% (Q17-18); 6.0 pp difference
- Synthesis methods – 82.9% (PRISMA item #13d) vs 91.5% (B,C); 8.6 pp difference
- Synthesis methods – 43.9% (PRISMA item #13f) vs 4.0% (F); 39.9 pp difference

The different results between fulfillment in PRISMA Checklist and our tool in items that were in theory similar is demonstrative of the difference between PRISMA’s “reporting transparency and completeness” and the “reported methodological quality” we were aiming to assess with our tool.

**Table 2. PRISMA 2020 Checklist and Reported Methodological Quality tool mapped frequency of fulfilled items.**

| **Reported Methodological Quality (RMQ) tool** | **PRISMA 2020 item** | **Fulfilled**  **RMQ tool** | **Fulfilled PRISMA 2020** |
| --- | --- | --- | --- |
| Q2. A review protocol exists and its registration information was available. | 24a | 22.0% | 22.0% |
|  | 24b |  |  |
| Q3. Inclusion criteria for primary studies were clearly defined. | 5 | 58.5% | 85.0% |
| Q4. No language-based exclusion criteria were defined. |  |  |  |
| Q5. Information sources (e.g., bibliographic databases) in the search were described. | 6 | 76.2% | 91.0% |
| Q6. Multiple electronic bibliographic databases were searched. |  |  |  |
| Q7. Electronic database searching was complemented with other information sources to identify relevant reports. |  |  |  |
| Q8. The identification of unpublished reports was explicitly made possible by the search. |  |  |  |
| Q9. The date last searched for information was described. |  |  |  |
| Q10. Full electronic search strategy was provided for at least one database. | 7 | 42.0% | 42.0% |
| Q11. Efforts were made to minimise error in selection of studies, namely by having more than one author independently participated in the study selection process. | 8 | 51.0% | 68.0% |
| Q12. Efforts were made to minimise error in data collection by having more than one author independently participating in the data extraction from reports. | 9 | 28.5% | 51.5% |
| Q13. Efforts were made to minimise error in data collection by using a prespecified form for data extraction from reports. |  |  |  |
| Q14. Processes for obtaining and confirming data from investigators were described. |  |  |  |
| Q15. Efforts to avoid double counting of participants/studies were described. |  |  |  |
| Q16. Variables for which data were sought were listed. | 10a | 63.0% | 63.0% |
|  | 10b |  |  |
| Q17. The risk of bias (or methodological quality) of individual studies was formally assessed using appropriate criteria. | 11 | 50.0% | 56.0% |
| Q18. Efforts were made to minimise error in risk of bias assessment, namely by having it independently conducted by at least two reviewers. |  |  |  |
| Q20. Methods used to assess certainty (or confidence) in the body of evidence for an outcome (e.g., by application of the GRADE framework) were described. | 15 | 9.0% | 9.0% |
| A. Meta-analytical summary measures were stated. | 12 | 85.4% | 85.4% |
| B. Meta-analytical models and methods were described. | 13d | 91.5% | 82.9% |
| C. Methods for assessing between-study variation (heterogeneity) and/or inconsistency were described. |  |  |  |
| D. Assessment of the risk of bias that may affect the cumulative evidence (publication bias or selective reporting) has been performed. | 14 | 51.2% | 51.2% |
| E. An intention to perform additional analyses has been reported (e.g., sensitivity or subgroup analyses, meta-regression) even if that not had been done on account of low heterogeneity or insufficient number of primary studies. | 13e | 43.9% | 43.9% |
| F. Biases in primary studies were addressed in meta-analysis. | 13f | 4.0% | 43.9% |

**References**

1. Page MJ, McKenzie JE, Bossuyt PM, et al. The PRISMA 2020 statement: an updated guideline for reporting systematic reviews. *BMJ*. Published online March 29, 2021:n71. doi:10.1136/bmj.n71

2. Moher D, Liberati A, Tetzlaff J, Altman DG. Preferred Reporting Items for Systematic Reviews and Meta-Analyses: The PRISMA Statement. *PLoS Med*. 2009;6(7):e1000097. doi:10.1371/journal.pmed.1000097
