# Supplementary material for: Reported methodological quality of medical systematic reviews: Development of an assessment tool (ReMarQ) and meta-research study
Source: Res Synth Methods. 2025 Mar 7;16(1):175–93. doi: 10.1017/rsm.2024.14 (PMC12621523; doi:10.1017/rsm.2024.14)
Supplement: Marques-Cruz et al. supplementary material 2 — Marques-Cruz et al. supplementary material [file S1759287924000140sup002.docx]

**Supplementary Tables**

**Supplementary Table 1. List of searched Journal Citation Reports categories and groups into which they were classified for this analysis**

| **Journal Citation Reports categories** | **Group*** |
| --- | --- |
| Allergy | G |
| Anesthesiology | G |
| Cardiac & Cardiovascular Systems | B |
| Clinical Neurology | F |
| Critical Care Medicine | A |
| Dentistry, Oral Surgery & Medicine | G |
| Dermatology | G |
| Emergency Medicine | A |
| Endocrinology & Metabolism | G |
| Gastroenterology & Hepatology | G |
| Genetics & Heredity | D |
| Gerontology | G |
| Hematology | B |
| Infectious Diseases | G |
| Medical Informatics | G |
| Medicine, General & Internal | G |
| Medicine, Legal | G |
| Medicine, Research & Experimental | D |
| Neuroimaging | F |
| Obstetrics & Gynecology | G |
| Oncology | C |
| Ophthalmology | G |
| Orthopedics | A |
| Otorhinolaryngology | G |
| Pathology | G |
| Pediatrics | E |
| Peripheral Vascular Disease | B |
| Pharmacology & Pharmacy | C |
| Primary Health Care | G |
| Psychiatry | G |
| Public, Environmental & Occupational Health | G |
| Radiology, Nuclear Medicine & Medical Imaging | F |
| Respiratory System | B |
| Rheumatology | G |
| Surgery | A |
| Transplantation | G |
| Tropical Medicine | G |
| Urology & Nephrology | G |

* - Class of selected article categories, resulting from a complete-linkage clustering.

Group A encompasses Critical Care Medicine, Emergency Medicine, Orthopedics and Surgery;

Group B encompasses Cardiac & Cardiovascular Systems, Hematology, Peripheral Vascular Disease and Respiratory System;

Group C encompasses Oncology and Pharmacology & Pharmacy;

Group D encompasses Genetics & Heredity and Medicine, Research & Experimental;

Group E encompasses Pediatrics;

Group F encompasses Clinical Neurology, Neuroimaging and Radiology, Nuclear Medicine & Medical Imaging;

Group G encompasses Allergy, Anesthesiology, Dentistry, Oral Surgery & Medicine, Dermatology, Endocrinology & Metabolism, Gastroenterology & Hepatology, Gerontology, Infectious Diseases, Medical Informatics, Medicine, General & Internal, Medicine, Legal, Obstetrics & Gynecology, Ophthalmology, Otorhinolaryngology, Pathology, Primary Health Care, Psychiatry, Public, Environmental & Occupational Health, Rheumatology, Transplantation, Tropical Medicine, Urology & Nephrology.

**Supplementary Table 2. Mapping of items of the reported methodological quality tool with the PRISMA 2009 and PRISMA 2020 checklist.**

| **Reported Methodological Quality tool** | **PRISMA 2009 checklist item [15]** | **PRISMA 2020 checklist item[10]** |
| --- | --- | --- |
| Q1. This systematic review claimed to comply with the PRISMA or other guidelines. |  |  |
| Q2. A review protocol exists and its registration information was available. | 5. Indicate if a review protocol exists, if and where it can be accessed (e.g., Web address), and, if available, provide registration information including registration number. | 24a. Provide registration information for the review, including register name and registration number, or state that the review was not registered. |
|  |  | 24b. Indicate where the review protocol can be accessed, or state that a protocol was not prepared. |
| Q3. Inclusion criteria for primary studies were clearly defined. | 6. Specify study characteristics (e.g., PICOS, length of follow-up) and report characteristics (e.g., years considered, language, publication status) used as criteria for eligibility, giving rationale. | 5. Specify the inclusion and exclusion criteria for the review and how studies were grouped for the syntheses. |
| Q4. No language-based exclusion criteria were defined. |  |  |
| Q5. Information sources (e.g., bibliographic databases) in the search were described. | 7. Describe all information sources (e.g., databases with dates of coverage, contact with study authors to identify additional studies) in the search and date last searched. | 6. Specify all databases, registers, websites, organisations, reference lists and other sources searched or consulted to identify studies. Specify the date when each source was last searched or consulted. |
| Q6. Multiple electronic bibliographic databases were searched. |  |  |
| Q7. Electronic database searching was complemented with other information sources to identify relevant reports. |  |  |
| Q8. The identification of unpublished reports was explicitly made possible by the search. |  |  |
| Q9. The date last searched for information was described. |  |  |
| Q10. Full electronic search strategy was provided for at least one database. | 8. Present full electronic search strategy for at least one database, including any limits used, such that it could be repeated. | 7. Present the full search strategies for all databases, registers and websites, including any filters and limits used. |
| Q11. Efforts were made to minimise error in selection of studies, namely by having more than one author independently participated in the study selection process. | 9. State the process for selecting studies (i.e., screening, eligibility, included in systematic review, and, if applicable, included in the meta-analysis). | 8. Specify the methods used to decide whether a study met the inclusion criteria of the review, including how many reviewers screened each record and each report retrieved, whether they worked independently, and if applicable, details of automation tools used in the process. |
| Q12. Efforts were made to minimise error in data collection by having more than one author independently participating in the data extraction from reports. | 10. Describe method of data extraction from reports (e.g., piloted forms, independently, in duplicate) and any processes for obtaining and confirming data from investigators. | 9. Specify the methods used to collect data from reports, including how many reviewers collected data from each report, whether they worked independently, any processes for obtaining or confirming data from study investigators, and if applicable, details of automation tools used in the process. |
| Q13. Efforts were made to minimise error in data collection by using a prespecified form for data extraction from reports. |  |  |
| Q14. Processes for obtaining and confirming data from investigators were described. |  |  |
| Q15. Efforts to avoid double counting of participants/studies were described. |  |  |
| Q16. Variables for which data were sought were listed. | 11. List and define all variables for which data were sought (e.g., PICOS, funding sources) and any assumptions and simplifications made. | 10a. List and define all outcomes for which data were sought. Specify whether all results that were compatible with each outcome domain in each study were sought (e.g. for all measures, time points, analyses), and if not, the methods used to decide which results to collect. |
|  |  | 10b. List and define all other variables for which data were sought (e.g. participant and intervention characteristics, funding sources). Describe any assumptions made about any missing or unclear information. |
| Q17. The risk of bias (or methodological quality) of individual studies was formally assessed using appropriate criteria. | 12. Describe methods used for assessing risk of bias of individual studies (including specification of whether this was done at the study or outcome level), and how this information is to be used in any data synthesis. | 11. Specify the methods used to assess risk of bias in the included studies, including details of the tool(s) used, how many reviewers assessed each study and whether they worked independently, and if applicable, details of automation tools used in the process. |
| Q18. Efforts were made to minimise error in risk of bias assessment, namely by having it independently conducted by at least two reviewers. |  |  |
| Q19. For procedures involving more than one reviewer, methods for solving disagreements were provided. |  |  |
| Q20. Methods used to assess certainty (or confidence) in the body of evidence for an outcome (e.g., by application of the GRADE framework) were described. |  | 15. Describe any methods used to assess certainty (or confidence) in the body of evidence for an outcome. |
| A. Meta-analytical summary measures were stated. ^a^ | 13. State the principal summary measures (e.g., risk ratio, difference in means). | 12. Specify for each outcome the effect measure(s) (e.g. risk ratio, mean difference) used in the synthesis or presentation of results. |
| B. Meta-analytical models and methods were described. ^a^ | 14. Describe the methods of handling data and combining results of studies, if done, including measures of consistency (e.g., *I*^2^) for each meta-analysis. | 13d. Describe any methods used to synthesize results and provide a rationale for the choice(s). If meta-analysis was performed, describe the model(s), method(s) to identify the presence and extent of statistical heterogeneity, and software package(s) used. |
| C. Methods for assessing between-study variation (heterogeneity) and/or inconsistency were described. ^a^ |  |  |
| D. Assessment of the risk of bias that may affect the cumulative evidence (publication bias or selective reporting) has been performed. ^a^ | 15. Specify any assessment of risk of bias that may affect the cumulative evidence (e.g., publication bias, selective reporting within studies). | 14. Describe any methods used to assess risk of bias due to missing results in a synthesis (arising from reporting biases). |
| E. An intention to perform additional analyses has been reported (e.g., sensitivity or subgroup analyses, meta-regression) even if that not had been done on account of low heterogeneity or insufficient number of primary studies. ^a^ | 16. Describe methods of additional analyses (e.g., sensitivity or subgroup analyses, meta-regression), if done, indicating which were pre-specified. | 13e. Describe any methods used to explore possible causes of heterogeneity among study results (e.g. subgroup analysis, meta-regression). |
| F. Biases in primary studies were addressed in meta-analysis. ^a^ |  | 13f. Describe any sensitivity analyses conducted to assess robustness of the synthesized results. |

a Solely assessed for systematic reviews with meta-analysis.

**Supplementary Table 3. Questions of the scale applied to assess the reported methodological quality of assessed systematic reviews, and their relation to the ROBIS signalling questions**

| **Reported Methodological Quality tool** | **ROBIS signalling questions [11]** |
| --- | --- |
| Q1. This systematic review claimed to comply with the PRISMA or other guidelines. |  |
| Q2. A review protocol exists and its registration information was available. | 4.2. Were all pre-defined analyses reported or departures explained? |
| Q3. Inclusion criteria for primary studies were clearly defined. |  |
| Q4. No language-based exclusion criteria were defined. | 2.4. Were restrictions based on date, publication format, or language appropriate? |
| Q5. Information sources (e.g., bibliographic databases) in the search were described. |  |
| Q6. Multiple electronic bibliographic databases were searched. | 2.1. Did the search include an appropriate range of databases/electronic sources for published and unpublished reports? |
| Q7. Electronic database searching was complemented with other information sources to identify relevant reports. | 2.2. Were methods additional to database searching used to identify relevant reports? |
| Q8. The identification of unpublished reports was explicitly made possible by the search. | 2.1. Did the search include an appropriate range of databases/electronic sources for published and unpublished reports? |
| Q9. The date last searched for information was described. |  |
| Q10. Full electronic search strategy was provided for at least one database. | 2.3. Were the terms and structure of the search strategy likely to retrieve as many eligible studies as possible? |
| Q11. Efforts were made to minimise error in selection of studies by having more than one author independently participated in the study selection process. | 2.5. Were efforts made to minimise error in selection of studies? |
| Q12. Efforts were made to minimise error in data collection by having more than one author independently participating in the data extraction from reports. | 3.1. Were efforts made to minimise error in data collection? |
| Q13. A prespecified form was used for data extraction from reports. |  |
| Q14. Processes for obtaining and confirming data from investigators were described. | 3.3. Were all relevant study results collected for use in the synthesis? |
| Q15. Efforts to avoid double counting of participants/studies were described. |  |
| Q16. Variables for which data were sought were listed. | 3.2. Were sufficient study characteristics available for both review authors and readers to be able to interpret the results? |
| Q17. The risk of bias (or methodological quality) of individual studies was formally assessed using appropriate criteria. | 3.4. Was risk of bias (or methodological quality) formally assessed using appropriate criteria? |
| Q18. Efforts were made to minimise error in risk of bias assessment, namely by having it independently conducted by at least two reviewers. | 3.5. Were efforts made to minimise error in risk of bias assessment? |
| Q19. For procedures involving more than one reviewer, methods for solving disagreements were provided. |  |
| Q20. Methods used to assess certainty (or confidence) in the body of evidence for an outcome (e.g., by application of the GRADE framework) were described. |  |
| A. Meta-analytical summary measures were stated. ^a^ |  |
| B. Meta-analytical model and methods were described. ^a^ |  |
| C. Methods for assessing between-study variation (heterogeneity) and/or inconsistency were described. ^a^ | 4.4. Was between-study variation (heterogeneity) minimal or addressed in the synthesis? |
| D. Assessment of the risk of bias that may affect the cumulative evidence (publication bias or selective reporting) has been performed. ^a^ | 4.5. Were the findings robust, e.g. as demonstrated through funnel plot or sensitivity analyses? |
| E. An intention to perform additional analyses has been reported (e.g., sensitivity or subgroup analyses, meta-regression) even if that not had been done on account of low heterogeneity or insufficient number of primary studies. ^a^ | 4.4. Was between-study variation (heterogeneity) minimal or addressed in the synthesis? |
|  | 4.5. Were the findings robust, e.g. as demonstrated through funnel plot or sensitivity analyses? |
| F. Biases in primary studies were addressed in meta-analysis. ^a^ | 4.6 Were biases in primary studies minimal or addressed in the synthesis? |

a Solely assessed for systematic reviews with meta-analysis.
